# Overdoses Involving Medetomidine Mixed with Opioids — Chicago, Illinois, May 2024

**DOI:** 10.15585/mmwr.mm7415a1

**Published:** 2025-05-01

**Authors:** Amy Nham, John N. Le, Shawn A. Thomas, Kimberly Gressick, Emily N. Ussery, Jean Y. Ko, R. Matt Gladden, Christina A. Mikosz, Joshua G. Schier, Alana Vivolo-Kantor, Maria Fiorillo, McKenna McMaster, Darlene Nolasco Magana, Livia Verklan-McInnes, Michael Wahl, Taylor Wood, Axel Adams, Alex Krotulski, Jordan Trecki, Ross Ellison, Roy Gerona, Ponni Arunkumar, Mojde Mir, Leslie M. Wise, Emma Betancourt, Kathleen Monty, Jhoanna Gulmatico, Angie Pojas, Ruchi Fitzgerald, Miao Hua

**Affiliations:** ^1^Epidemic Intelligence Service, CDC; ^2^Chicago Department of Public Health, Chicago, Illinois; ^3^National Center for HIV, Viral Hepatitis, STD, and TB Prevention, CDC; ^4^National Center on Birth Defects and Developmental Disabilities, CDC; ^5^National Center for Injury Prevention and Control, CDC; ^6^Illinois Poison Center, Chicago, Illinois; ^7^Chicago Recovery Alliance, Chicago, Illinois; ^8^University of Illinois College of Medicine, Chicago, Illinois; ^9^The Center for Forensic Science Research & Education, Horsham, Pennsylvania; ^10^Drug Enforcement Administration, Arlington, Virginia; ^11^University of California San Francisco, San Francisco, California; ^12^Cook County Medical Examiner, Chicago, Illinois; ^13^Illinois Department of Public Health; ^14^Oak Park Public Health Department, Oak Park, Illinois; ^15^Mount Sinai Hospital, Chicago, Illinois; ^16^Humboldt Park Health, Chicago, Illinois; ^17^West Suburban Medical Center, Oak Park, Illinois.

**Summary**

**What is already known about this topic?**

Medetomidine, a nonopioid sedative not approved for use in humans, has been detected in illegally manufactured opioids across North America since 2022.

**What is added by this report?**

Twelve confirmed and 26 probable cases of medetomidine-involved overdose occurred in Chicago, Illinois, during May 11–17, 2024, mostly among non-Hispanic Black or African American men aged 45–64 years. Bradycardia and lack of response to naloxone were defining clinical features. Fentanyl was present in all blood specimens and drug samples that tested positive for medetomidine.

**What are the implications for public health practice?**

Multisector surveillance is needed to quickly identify and respond to new adulterants introduced into the illegal drug supply. Clinicians who observe atypical toxidromes associated with suspected opioid-involved overdoses should contact their local health department and continue to provide naloxone and linkage to evidence-based treatment.

## Abstract

Medetomidine, a nonopioid sedative not approved for use in humans, has periodically been detected in illegally manufactured opioids across North America since 2022. On May 11, 2024, the Chicago Department of Public Health (CDPH) and the Illinois Department of Public Health were alerted by hospitals and the Illinois Poison Center to an increase in emergency medical services responses for suspected opioid-involved overdoses with atypical symptoms, mostly clustered on Chicago’s West Side. CDPH and CDC investigated and identified 12 confirmed, 26 probable, and 140 suspected overdoses involving medetomidine mixed with opioids among patients treated at three hospitals in Chicago’s West Side during May 11–17, 2024. Medetomidine had not been previously identified in Chicago’s illegal drug supply. Fentanyl was identified in all drug samples and blood specimens containing medetomidine. Most patients were male, non-Hispanic Black or African American, and aged 45–64 years; most patients with confirmed cases experienced bradycardia and had no or only a partial response to naloxone. This cluster is the largest reported for confirmed medetomidine-involved overdoses. Multisector surveillance, including by health care providers, toxicology laboratories, and public health personnel, was essential for quickly identifying and responding to new adulterants in the illegal drug supply. Because all specimens and samples in this investigation that contained medetomidine also contained natural or synthetic opioids, administering naloxone for all suspected opioid-involved overdoses remains crucial.

## Introduction

On May 11, 2024, the Chicago Department of Public Health (CDPH) and the Illinois Department of Public Health (IDPH) were alerted by the Overdose Detection Mapping Application Program^†^ that 50 emergency medical services (EMS) responses for suspected opioid-involved overdoses occurred that day, a number more than two standard deviations above the 2023 daily average (27.4) in Chicago. Events were mostly clustered on Chicago’s West Side. Area hospitals and the Illinois Poison Center (IPC) also notified CDPH of several patients observed with bradycardia and suspected opioid-involved overdose symptoms not fully reversed by naloxone during the weekend of May 11.

## Methods

Initial toxicologic tests from samples of bagged powders in the possession of five patients in the emergency department (ED) detected medetomidine mixed with fentanyl, in varying concentrations and ratios. Medetomidine, a central nervous system depressant not approved for use in humans and potentially more potent than xylazine (*1*), had recently appeared as an adulterant in the national illegal drug supply (*2*); this medetomidine detection represented the first detection in Chicago.

On May 17, CDPH requested CDC assistance to investigate the suspected opioid-involved overdose cluster. The investigation used four data sources for analysis: 1) blood specimen and drug sample results sent by hospitals on the advice of IPC to the Drug Enforcement Administration’s Toxicology Testing Program’s (DEA TOX) contract laboratory at University of California, San Francisco, and the Center for Forensic Science Research and Education with the assistance of the Chicago Recovery Alliance, 2) mortality data from the Cook County medical examiner’s office, 3) EMS records from the Chicago Fire Department, and 4) medical records from three EDs on Chicago’s West Side that received the most patients from suspected opioid-involved overdose EMS responses during May 11–17, 2024.

Using these data sources, a case identification algorithm was developed defining patients as having a confirmed, probable, or suspected overdose involving medetomidine mixed with opioids. A confirmed case was defined as a case in a patient treated for suspected opioid-involved overdose^§^ whose blood specimen tested positive for medetomidine. A probable case was defined as a case in a patient who 1) possessed a drug sample containing medetomidine or 2) experienced bradycardia (heart rate less than 60 beats per minute) with symptoms not fully reversed by naloxone (defined as persistent altered mental status after naloxone administration) during the EMS response or upon ED arrival. Suspected cases were all other suspected opioid-involved overdoses among patients who were admitted to one of the three hospital EDs, even without clinical or testing evidence for medetomidine, because the patients were admitted during a time of medetomidine infiltration of the drug supply. A patient was considered to not have a case of overdose involving medetomidine mixed with opioids if their blood specimen tested negative for medetomidine.

Demographic characteristics, clinical signs and symptoms, and clinical course were abstracted from medical charts for confirmed and probable cases. Partial chart abstractions^¶^ were completed for suspected cases. Descriptive data were managed and analyzed using SAS software (version 9.4; SAS Institute). This activity was reviewed by CDC, deemed not research, and was conducted consistent with applicable federal law and CDC policy.**

## Results

**FIGURE 1:**
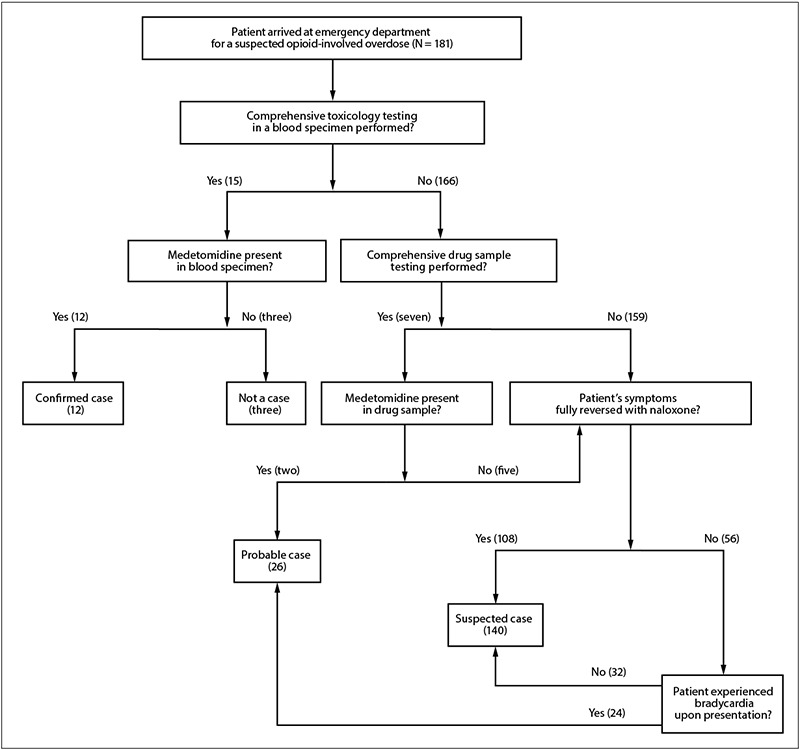
Case identification algorithm used for patients admitted to three emergency departments for overdoses involving medetomidine mixed with opioids — Chicago, Illinois, May 11–17, 2024*^,^^†^^,^^§^^,^^¶^^,^**^,^^††^ * Three patients for whom comprehensive toxicology testing of blood specimens was performed also had drug sample testing; medetomidine was present in the blood specimens and drug samples from all three patients. ^†^ For the patient blood specimens in which medetomidine was detected, diphenhydramine (12 patients), fentanyl (12), quinine (11), benzoylecgonine (a cocaine metabolite) (10), bromazolam (a benzodiazepine) (six), morphine (six), and xylazine (six) were also detected. ^§^ For the patients for whom medetomidine was present in drug samples, the drug samples were bagged powders that were in the patients’ possession at the initial health care contact. ^¶^ A patient was classified as not having symptoms fully reversed with naloxone if the patient experienced persistent altered mental status after naloxone administration. ** Suspected cases were defined as other suspected opioid-involved overdoses in patients who were admitted to the emergency departments of the three hospitals even without clinical or testing evidence for medetomidine because the patients were admitted during a time of medetomidine infiltration of the drug supply. ^††^ Bradycardia was defined as heart rate less than 60 beats per minute.

**FIGURE 2:**
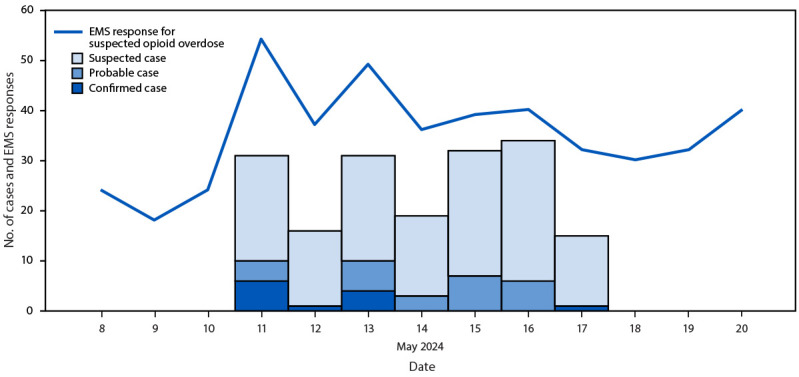
Overdose cases involving medetomidine mixed with opioids and emergency medical services responses* for suspected opioid-involved overdose cases per day^†^ — Chicago, Illinois, May 8–20, 2024^§^ **Abbreviation:** EMS = emergency medical services. * EMS responses data are from the Chicago Fire Department. Numbers might differ from near real-time EMS response data from the Office of National Drug Control Policy's Overdose Detection Mapping Application Program, which links first responders and records management systems to a mapping tool to track overdoses. The finalized Chicago Fire Department data indicated that there were 54 opioid-related EMS responses on May 11, 2024. ^†^ Twelve confirmed, 26 probable, and 140 suspected medetomidine-involved overdose cases were identified. ^§^ During May 11–17, total EMS responses for suspected opioid-involved overdoses included confirmed, probable, and suspected cases of medetomidine-involved overdose. Suspected cases were defined as suspected opioid-involved overdoses in patients who were admitted to the emergency departments of the three hospitals even without clinical or testing evidence for medetomidine because the patients were admitted during a time of medetomidine infiltration of the drug supply.

Among 181 patients treated for suspected opioid-involved overdose at the three EDs during May 11–17, CDPH identified 12 confirmed, 26 probable, and 140 suspected cases; three patients were determined to not have experienced a medetomidine-involved overdose (Figure 1) (Figure 2).

Confirmed and probable cases were identified using results from 15 blood specimens from unique patients and 10 drug samples from unique patients sent for testing; three patients had both blood specimens and drug samples sent for testing (Figure 1). Among the 15 blood specimens, 12 tested positive for medetomidine in combination with the following substances: diphenhydramine (12 patients), fentanyl (12), quinine (11), benzoylecgonine (10),^††^ morphine (six),^§§^ xylazine (six),^¶¶^ and bromazolam (six).*** Among the 10 drug samples, five tested positive for medetomidine and fentanyl,^†††^ and among these five, medetomidine was also present in corresponding blood specimens for three. Thus, drug sample testing identified two additional probable cases; the remaining 24 probable cases were identified using clinical data.

The 38 confirmed and probable cases were among mostly male persons (84%), non-Hispanic Black or African American persons (87%), and persons aged 45–64 years (63%) (Table). Among all 38 confirmed and probable cases, 18 (47%) patients reported heroin as the intended drug of use at the time of overdose. Snorting was the most common route of administration, reported by eight (21%) patients. However, the drugs that patients intended to use and route of administration were unknown for most patients. The most common clinical signs and symptoms were hypertension (36; 95%), bradycardia (33; 87%), altered mental status (32; 84%), pinpoint pupils (32; 84%), and hypoxemia with blood oxygen saturation <90% (18; 47%). Five persons required treatment with atropine, a first-line medication for the treatment of bradycardia. Elevated systolic blood pressure ≥180 mm Hg was observed in 16 (42%) patients. Among 12 confirmed cases, 11 (92%) patients experienced partial or no improvement of symptoms after naloxone administration. One patient had full reversal of symptoms; this patient also had the lowest serum concentration of medetomidine among those with blood specimen results. Blood medetomidine concentrations ranged from 0.7 ng/mL to 63.7 ng/mL.

**TABLE:** Demographic and clinical characteristics of patients with confirmed, probable, and suspected cases of overdose involving medetomidine mixed with opioids — Chicago, Illinois, May 11–17, 2024

Characteristic	No. (%)			
	Confirmed (n = 12)	Probable (n = 26)	Suspected* (n = 140)	Total (N = 178)
**Median age, yrs (range)**	59.8 (38.2–69.6)	59.1 (32.5–86.5)	54.9 (22.1–78.7)	**55.8 (22.1–86.5)**
**Age group, yrs**				
<34	0	1 (3.8)	19 (13.6)	**20 (11.2)**
35–44	2 (16.7)	2 (7.7)	20 (14.3)	**24 (13.5)**
45–64	7 (58.3)	17 (65.4)	80 (57.1)	**104 (58.4)**
≥65	3 (25.0)	6 (23.1)	21 (15.0)	**30 (16.9)**
**Sex**				
Female	2 (16.7)	4 (15.4)	29 (20.7)	**35 (19.7)**
Male	10 (83.3)	22 (84.6)	111 (79.3)	**143 (80.3)**
**Race and ethnicity^†^**				
Black or African American	10 (83.3)	23 (88.5)	97 (69.3)	**130 (73.0)**
White	2 (16.7)	0	15 (10.7)	**17 (9.6)**
Hispanic or Latino	0	1 (3.8)	8 (5.7)	**9 (5.1)**
Other	0	1 (3.8)	11 (7.9)	**12 (6.7)**
Unknown	0	1 (3.8)	9 (6.4)	**10 (5.6)**
**History of substance use**				
Yes	10 (83.3)	16 (61.5)	—	**26 (68.4)**
No	0	2 (7.7)	—	**2 (5.3)**
Unknown	2 (16.7)	8 (30.8)	—	**10 (26.3)**
**Reported drug used immediately before overdose event** ^§^				
Heroin	5 (41.7)	13 (50.0)	—	**18 (47.4)**
Other opiate	1 (8.3)	2 (7.7)	—	**3 (7.9)**
Unknown	6 (50.0)	11 (42.3)	—	**17 (44.7)**
**Route of drug used before overdose event**				
Snorting	4 (33.3)	4 (15.4)	—	**8 (21.1)**
Unknown	8 (66.7)	22 (84.6)	—	**30 (78.9)**
**Response to naloxone** ^¶^				
Full reversal of symptoms	1 (8.3)	0	108 (77.1)	**109 (61.2)**
Partial improvement of symptoms**	7 (58.3)	19 (73.1)	19 (13.6)	**45 (25.3)**
No improvement of symptoms	4 (33.3)	6 (23.1)	5 (3.6)	**15 (8.4)**
Naloxone given but response not documented	0	0	1 (0.7)	**1 (0.6)**
Naloxone not given	0	1 (3.8)	6 (4.3)	**7 (3.9)**
**Sign or symptom^§,††,§§^**				
Hypertension	12 (100)	24 (92.3)	100 (71.4)	**136 (76.4)**
Bradycardia	9 (75.0)	24 (92.3)	23 (16.4)	**56 (31.5)**
Pinpoint pupils	10 (83.3)	22 (84.6)	86 (61.4)	**118 (66.3)**
Altered mental status	12 (100)	20 (76.9)	82 (58.6)	**114 (64.0)**
Hypoxemia	7 (58.3)	11 (42.3)	27 (19.3)	**45 (25.3)**
Systolic blood pressure ≥180 mm Hg	8 (66.7)	8 (30.8)	35 (25.0)	**51 (28.7)**
Bradypnea	2 (16.7)	6 (23.1)	20 (14.3)	**28 (15.7)**
Downward gaze	3 (25.0)	0	0	**3 (1.7)**
Twitching	1 (8.3)	0	1 (0.7)	**2 (1.1)**
Apnea	0	5 (19.2)	4 (2.9)	**9 (5.1)**
Hypotension	0	3 (11.5)	3 (2.1)	**6 (3.4)**
Dilated pupils	0	0	1 (0.7)	**1 (0.6)**
Hypothermia	0	0	1 (0.7)	**1 (0.6)**
**Admitted to hospital**				
Yes	8 (66.7)	8 (30.8)	26 (18.6)	**42 (23.6)**
Yes, to intensive care unit^¶¶^	5 (62.5)	4 (50.0)	9 (34.6)	**18 (42.9)**
Length of inpatient stay, median days (range)	3.5 (0.4–5.8)	2.0 (0.5–2.7)	1.2 (0.1–5.0)	**1.9 (0.1–5.8)**
No	4 (33.3)	18 (69.2)	113 (80.7)	**135 (75.8)**
**Received medications for opioid use disorder**				
Yes	9 (75.0)	6 (23.1)	—	**17 (44.7)**
No	3 (25.0)	20 (76.9)	—	**24 (63.2)**
**Received atropine**				
Yes	3 (25.0)	2 (7.7)	—	**5 (13.2)**
No	9 (75.0)	24 (92.3)	—	**33 (86.8)**
**Received respiratory support**				
Yes^§^	7 (58.3)	9 (34.6)	—	**16 (42.1)**
Supplemental oxygen***	6 (85.7)	6 (66.7)	—	**12 (75)**
Continuous positive airway pressure***	0	1 (11.1)	—	**1 (6.25)**
Intubation***	3 (42.9)	2 (22.2)	—	**5 (31.3)**
No	5 (41.7)	17 (65.4)	—	**22 (57.9)**
**Disposition after discharge**				
Home	8 (66.7)	25 (96.2)	120 (85.7)	**153 (86.0)**
Left against medical advice	3 (25.0)	0	18 (12.9)	**21 (11.8)**
Referral to other health care facility	1 (8.3)	1 (3.8)	0	**2 (1.1)**
Deceased	0	0	1 (0.7)	**1 (0.6)**
Unknown	0	0	1 (0.7)	**1 (0.6)**
**Linkage to care and harm reduction resources^§^**				
Provided patient naloxone	5 (41.7)	12 (46.2)	—	**17 (44.7)**
Provided patient informational resources	1 (8.3)	2 (7.7)	—	**3 (7.9)**
Referral to behavioral health treatment for substance use disorder	8 (66.7)	11 (42.3)	—	**19 (50.0)**
Prescribed medications for opioid use disorder	3 (25.0)	1 (3.8)	—	**4 (10.5)**
Referral to treatment for other comorbidities	0	1 (3.8)	—	**1 (2.6)**
None	0	3 (11.5)	—	**3 (7.9)**
Unknown	0	1 (3.8)	—	**1 (2.6)**

* Time and resource constraints resulted in the completion of only partial chart abstractions for suspected cases, and data were not collected for all variables; data not collected are indicated with a dash.

^†^ Persons of Hispanic or Latino (Hispanic) origin might be of any race but are categorized as Hispanic; all racial groups are non-Hispanic.

^§^ Not mutually exclusive.

^¶^ Differences in response to naloxone are partially attributable to case definitions of probable and suspected cases.

** Partial response to naloxone is defined as a patient having some remaining opioid-involved overdose symptoms (i.e., prolonged altered mental status) after naloxone administration.

^††^ Ordered by descending frequency of symptoms among confirmed and probable cases combined; if frequency was equal among confirmed and probable cases, confirmed ranked above probable.

^§§^ Signs and symptoms were based on emergency medical services or vital signs data collected on arrival at the hospital or if noted in the medical record. Criteria were 1) systolic blood pressure ≥140 mm Hg for hypertension; 2) heart rate less than 60 beats per minute or demonstration on electrocardiogram for bradycardia (differences in bradycardia are partially attributable to the case definitions of probable and suspected cases); 3) respiratory rate less than 12 breaths per minute for bradypnea; 4) systolic blood pressure ≥180 mm Hg for hypertensive urgency; 5) oxygen saturation level <90% for hypoxemia; and 6) systolic blood pressure <90 mm Hg for hypotension.

^¶¶^ Among patients who were hospitalized.

*** Among patients who received respiratory support.

Among the 38 patients with confirmed and probable cases, 16 were admitted to the hospital, nine required admission to an intensive care unit, 16 received respiratory support, and five required intubation. One death in a patient with a suspected opioid overdose was classified as a suspected medetomidine-affected overdose case; however, in the absence of toxicologic confirmation, the death was not definitively linked to medetomidine.

## Public Health Response

On May 14, CDPH released a health alert^§§§^ describing the increase in EMS responses for suspected opioid-involved overdoses during the weekend of May 11. After medetomidine was detected in multiple drug samples, CDPH released a second health alert^¶¶¶^ on May 20. Medical and public health personnel were advised to inform IPC of suspected opioid-involved overdoses that appeared atypical, report overdose clusters at a single facility to CDPH, and pursue toxicology testing through programs such as DEA TOX,**** which tests biologic specimens from patients who experience drug overdoses for new psychoactive substances. On May 21, IDPH expanded the health alert statewide.

CDPH collaborated with partners to promote community-based point-of-care drug checking^††††^ and monthly reporting in areas with the highest number of EMS transports for suspected opioid-involved overdoses. CDPH also advised EDs that treat the highest numbers of suspected opioid-involved overdoses on recommended protocols and practices for predischarge administration of medications for opioid use disorder and linking patients to care.

## Discussion

Although medetomidine has periodically been detected in multiple states and in Canada since 2022, this report is the first to characterize demographic and clinical characteristics of a cluster of overdoses involving medetomidine mixed with opioids (*2*–*4*). Efficient collaboration across sectors, including health care, toxicology laboratories, and public health, was essential in identifying and swiftly responding to the emergence of medetomidine in Chicago’s illegal drug supply. Comprehensive toxicology testing initiated by hospitals, their reports to IPC, and timely coordination and testing of drug samples and blood specimens identified medetomidine as the contributing factor of the overdose cluster.

Since 2023, CDC has supported toxicologic testing of illegal drug paraphernalia or samples in 18 local jurisdictions (including Chicago) (*5*). Additional analyses of data collected can provide states and local jurisdictions with critical information to lessen the public health risks caused by changes in the illegal drug market, including introduction of new drugs or adulterants like medetomidine, that can increase the risk for overdose or other negative outcomes.

The emergence of medetomidine in the illegal drug supply can complicate responses to suspected opioid-involved overdoses and necessitates educating persons who use drugs, clinicians, and public health personnel about the adverse effects of medetomidine. Bradycardia, a side effect typically more intense with medetomidine than with opioids, was observed frequently in this investigation and might help to clinically distinguish overdoses involving medetomidine mixed with opioids from those involving only opioids. Hypertensive urgency was also observed. Cardiovascular and respiratory support are crucial to the management of medetomidine toxicity (*6*). Although peripheral vasoconstriction caused by medetomidine has been described in animals, whether medetomidine exacerbates skin and soft tissue damage that is associated with xylazine in humans remains unclear (*7*).

Clinicians who observe an atypical toxidrome associated with a suspected opioid-involved overdose should administer naloxone and provide supportive care and should have a low threshold for contacting their local health department, especially regarding clusters of overdoses with atypical, similar toxidromes. Poison centers can provide clinical guidance on patient care and assist with coordination of toxicology testing.

All blood specimens and drug samples in this investigation that contained medetomidine also contained natural or synthetic opioids, the effects of which are reversible with naloxone. Despite the emergence of new adulterants, administering naloxone for all suspected opioid-involved overdoses remains important, including for those overdoses involving medetomidine mixed with opioids. The effects of medetomidine cannot be reversed with naloxone. In addition, the antidote for medetomidine and dexmedetomidine, atipamezole, is not approved for use in humans (*8*). Clinicians should continue to provide medications for opioid use disorder, linkage to care, and harm reduction services for persons experiencing opioid use disorder (*9*).

### Limitations

The findings in this report are subject to at least four limitations. First, suspected cases might have been overestimated because no clear toxidrome for medetomidine-involved overdoses exists, a result of minimal published data on its effects in humans. For the purposes of the investigation, sensitivity was prioritized over specificity for suspected cases during this period so as to not miss any potential cases in patients with increased risk for medetomidine exposure. Second, most patients had no toxicology testing, which might have contributed to an underestimation of confirmed cases. Because medetomidine is an emerging adulterant, it is not part of standard urine drug screening. Testing for medetomidine requires sending specimens and samples to a specialized toxicology laboratory, increasing the barrier for identifying cases. Third, time and resource constraints limited the investigation to three hospitals; local hospitals not included in the study might have received patients with overdoses involving medetomidine. Therefore, these findings might not be generalizable. Finally, the investigation was conducted during May 11–17, 2024, and additional cases might have occurred outside of this time frame. No additional clusters attributable to medetomidine have since been identified in Chicago; however, additional drug samples obtained since that time have tested positive for medetomidine.^§§§§^

### Implications for Public Health Practice

This cluster of confirmed medetomidine-involved overdoses is the largest yet reported, and the landscape of adulterants in the illegal drug supply is ever-changing and expanding. The recent addition of xylazine has led to a concerning trend in deaths potentially resulting from adulteration in the fentanyl supply (*10*), and the emergence of medetomidine further complicates the opioid overdose crisis. Clinicians and persons who use illegal drugs should be aware that medetomidine can be present in the drug supply. Although medetomidine effects cannot be reversed with naloxone, if a person might be overdosing, the use of naloxone or any other opioid overdose reversal medication is recommended. In addition, connecting persons at risk for overdose to evidence-based treatment, services, and support can save lives.

## Footnotes

* These authors contributed equally to this report.

† The program, developed by the Office of National Drug Control Policy, links first responders and records management systems to a mapping tool to track overdoses and stimulate real-time response and strategic analysis across jurisdictions. ODMAP

§ *International Classification of Diseases, Tenth Revision* codes and chief complaints for suspected opioid-involved overdoses were identified on the basis of the CDC definition for ED visits. CDC All Opioid Overdose v4 Parsed

¶ Partial chart abstractions included demographic information, naloxone doses received, response to naloxone, patient symptoms and vital signs, and hospital admission and disposition. Full chart abstractions included the partial chart data as well as substance use history, underlying health conditions and medications, overdose event details, hospital treatments, complications, and linkage to care. Not all data obtained are included in this report.

** 45 C.F.R. part 46.102(l)(2), 21 C.F.R. part 56; 42 U.S.C. Sect. 241(d); 5 U.S.C. Sect. 552a; 44 U.S.C. Sect. 3501 et seq.

†† Benzoylecgonine is a cocaine metabolite.

§§ Morphine is an opioid agonist and also a metabolite of heroin and codeine.

¶¶ Xylazine is a nonscheduled, nonopioid sedative not approved for human use and has been increasingly detected in illegal opioids.

*** Bromazolam is a benzodiazepine.

††† Three of the five drug samples had a similar composition of medetomidine in the highest concentration, followed by diphenhydramine, heroin, and trace amounts of fentanyl. Xylazine was detected in two of the five samples, having higher concentrations of xylazine than medetomidine. One of the five contained trace amounts of nitazenes (metonitazene and *N*-pyrrolidino-metonitazene), synthetic opioids more potent than fentanyl.

§§§
Increase in Opioid Overdoses May 14, 2024

¶¶¶
Medetomidine in Chicago’s Drug Supply May 20, 2024

****
DEA TOX Toxicology Testing Program

††††
Drug Checking Programs in the United States and Internationally: Environmental Scan Summary

§§§§
Overdose Cluster Associated with Nitazenes and the Novel Adulterant BTMPS November 8, 2024
